# Renal imaging at 5 T versus 3 T: a comparison study

**DOI:** 10.1186/s13244-022-01290-9

**Published:** 2022-09-24

**Authors:** Liyun Zheng, Chun Yang, Ruofan Sheng, Yongming Dai, Mengsu Zeng

**Affiliations:** 1 Shanghai Institute of Medical Imaging, Shanghai, China; 2 Department of Radiology, Zhongshan Hospital, Fudan University, No. 180 Fenglin Road, Xuhui District, Shanghai, 20032 China; 3 Shenzhen United Imaging Research Institute of Innovative Medical Equipment, Shenzhen, China; 4 MR Collaboration, Central Research Institute, United Imaging Healthcare, 2258 Chengbei Rd., Jiading District, Shanghai, 201807 China

**Keywords:** High-field MRI, Abdominal imaging, Renal MRI, Anatomical imaging, Functional imaging

## Abstract

**Background:**

Recently, a whole-body 5 T MRI scanner was developed to open the door of abdominal imaging at high-field strength. This prospective study aimed to evaluate the feasibility of renal imaging at 5 T and compare the image quality, potential artifacts, and contrast ratios with 3 T.

**Methods:**

Forty healthy volunteers underwent MRI examination both at 3 T and 5 T. MRI sequences included T1-weighted gradient-echo (GRE), T2-weighted fast spin echo, diffusion-weighted imaging, and multi-echo GRE T2* mapping. Image quality and presence of artifacts were assessed for all sequences using four-point scales. For anatomical imaging, the signal-to-noise ratio (SNR) and contrast ratio (CR) of abdomen organ tissues were calculated. Besides, for functional imaging, the contrast-to-noise ratio of cortex/medulla was calculated. Wilcoxon signed rank-sum test was used to compare the visual evaluation scores and quantitative measurements between 3 and 5 T images.

**Results:**

Compared to 3 T examination, T1-weighted sequence at 5 T showed significantly better image quality with higher conspicuity of the renal veins and arteries, and comparable artifacts. Image quality was comparable between both field strengths on T2-weighted images, whereas a significantly higher level of artifacts was observed at 5 T. Besides, 5 T MRI contributed to higher SNR and CR for abdomen organ tissues. For functional imaging, 5 T MRI showed improved corticomedullar discrimination. There was no significant difference between apparent diffusion coefficient of renal at 3 T and 5 T, while 5 T MRI resulted in significantly shorter T2* values in both cortex and medulla.

**Conclusions:**

5 T MRI provides anatomical and functional images of the kidney with sufficient image quality.

## Key points

- Anatomical and functional renal MRI at 5 T had sufficient image quality.
- 5 T MRI demonstrated higher conspicuity of the renal vasculature than 3 T MRI.
- 5 T MRI contributed to improved corticomedullar discrimination than 3 T MRI.
- Renal DWI and T2* map were found to be feasible at 5 T.

## Background

Motivated by the promise of higher signal-to-noise ratio (SNR), increased resolution and/or reduced imaging time, new or better tissue contrast, and improved parallel imaging performance, human magnetic resonance imaging (MRI) at higher magnetic fields (> 3 T) has been a major research focus in recent years [1–3]. MRI at ultra-high magnetic fields (7 T) demonstrated intriguing capabilities and benefits for neuroradiological imaging [4, 5] and for assessing degenerative joint diseases of the musculoskeletal system [6, 7]. Based on prior research works, the first regulatory approval of a commercial 7 T MRI system for clinical neuro and musculoskeletal imaging as a medical device occurred in 2017 [8, 9].

Meanwhile, MRI at ultra-high field encounters numerous challenges, especially in abdominal imaging. The two main challenges of 7 T MRI are transmit B1 + inhomogeneities in large body cross sections and radiofrequency (RF) power deposition in tissue [10]. The abdominal imaging applications at 7 T are known to be impaired due to the short wavelength of the 298 MHz RF field and enhanced tissue absorption, leading to strong flip angle variations and limited penetration depth [11]. In recent years, methodological developments regarding RF transmit strategies and specific absorption rate (SAR) supervision were enabling the exploitation of the potential of body imaging applications at 7 T. For instance, Laader et al. demonstrated the feasibility and overall comparable imaging ability of T1-weighted 7 Tesla abdominal MRI toward 3 Tesla and 1.5 Tesla MRI, yielding a promising diagnostic potential for non-enhanced magnetic resonance angiography (MRA) [12]. Umutlu et al. demonstrated the feasibility and diagnostic potential of dedicated 7 T renal imaging [13]. Nevertheless, according to the prior study, 1.5 T and 3 T MRI offered comparably high-quality T2-weighted abdominal imaging, showing superior diagnostic quality over 7 T MRI [12]. Besides, the T2-weighted fast spin echo (FSE) sequence of renal MRI at 7 T was proved to be strongly impaired because of signal heterogeneities [13].

Recently, a whole-body 5 T MRI scanner was developed not only to inherit higher SNR and spatial resolution in neurology and orthopedics at 7 T but to open the door of abdominal imaging at field strength beyond 3 T [14]. In this prospective study, the aim was to investigate the feasibility of abdominal imaging at 5 T, compared to conventional 3 T in respect of SNR, contrast-to-noise ratio (CNR), imaging artifacts, and image quality. More specifically, the performance of structural and functional renal MRI at 5 T was evaluated.

## Methods

### Participants

This prospective study was approved by the institutional review board, and written signed consents were obtained from all participants before each examination. All participants were informed about the potential risks associated with high-field MRI, including mild nausea, vertigo, headache, tingling, and tapping sensations because of peripheral nerve stimulation [15]. The inclusion criteria were as follows: (a) healthy subjects in the absence of any clinical history of renal diseases including renal stones; (b) age between 18 and 65 years old. The exclusion criteria were as follows: (a) hypertension (systolic or diastolic pressure ≥ 140/90 mmHg) or hypotension (systolic blood pressure < 85 mmHg); (b) pregnancy (as determined by urine pregnancy test); (c) abnormal findings of the kidney on MRI; (d) any contraindication to MRI; and (e) incomplete MRI examination.

### MRI acquisitions

For each subject, MRI examination was performed with a 3.0-Tesla MRI scanner (uMR 790, United Imaging Healthcare, Shanghai, China) and a 5.0-Tesla MRI scanner (uMR Jupiter, United Imaging Healthcare, Shanghai, China). A custom-built 24-channel body coil was used for all studies at 5 T using local B1 + shimming for B1 + optimization. Following MR sequences were acquired: a. transverse breath-hold T1-weighted volume interpolated gradient-echo sequence (QUICK 3D) with fat suppression; b. coronal breath-hold T1-weighted QUICK 3D with fat suppression; c. transverse T2-weighted fat-saturated FSE sequence with respiratory trigger. The detailed MR protocols for anatomical imaging are listed in Table 1.

**Table 1:** MRI protocols for anatomical imaging at 3 T and 5 T

Parameters	Sequences					
	Axial T1W 3D GRE		Coronal T1W 3D GRE		Axial T2W FSE	
	3 T	5 T	3 T	5 T	3 T	5 T
TR (ms)	3.49	3.49	3.84	3.84	*	*
TE (ms)	1.48	1.45	1.65	1.60	88.00	83.52
FA (°)	15	20	15	25	130	130
FOV (mm × mm)	300 × 400	300 × 400	380 × 380	380 × 380	300 × 380	300 × 380
Matrix	408 × 544	408 × 544	456 × 456	456 × 456	306 × 456	306 × 456
ST (mm)	3	3	3	3	6	6
Number of slices	66	66	64	64	24	24
Bandwidth (Hz/pixel)	700	700	600	600	260	260

^*^TR depends on the respiratory interval of participants

*MRI* magnetic resonance imaging; *T1W* T1-weighted; *T2W* T2-weighted; *GRE* gradient-echo; *FSE* fast spin echo; *TR* repetition time; *TE* echo time; *FA* flip angle; *FOV* field of view; *ST* slice thickness

For functional imaging, echo-planar imaging diffusion-weighted imaging (EPI—DWI) with reduced FOV was acquired. Besides, renal T2* mapping was performed based on multi-echo GRE sequence. The detailed MR protocols for functional imaging are listed in Table 2.

**Table 2:** MRI protocols for functional imaging at 3 T and 5 T

Parameters	Sequences			
	EPI-DWI		T2* mapping	
	3 T	5 T	3 T	5 T
TR (ms)	*	*	181.80	202.20
TE (ms)	89.30	65.60	2.29, 5.56, 8.83, 12.10, 15.37	3.67, 6.98, 10.29, 13.60, 16.91
FA (°)	90	90	60	60
FOV (mm)	120 × 280	120 × 280	300 × 400	300 × 400
Matrix	246 × 576	246 × 576	154 × 256	154 × 256
ST (mm)	4	4	5	5
Number of slices	24	24	28	28
Bandwidth (Hz/pixel)	1660	1510	400	400
b-value (s/mm [2])	0, 50, 800	0, 50, 800	/	/

^*^TR depends on the respiratory interval of participants

*MRI* magnetic resonance imaging; *EPI* echo-planar imaging; *DWI* diffusion-weighted imaging; *TR* repetition time; *TE* echo time; *FA* flip angle; *FOV* field of view; *ST* slice thickness

### Qualitative image analysis

All the qualitative analyses were performed by two experienced radiologists (C.Y., with more than 14-years’ experience of abdominal MRI; M.Z., with more than 16-years’ experience of abdominal MRI) blindly and independently. For the anatomical imaging, the overall image quality was evaluated based on the corticomedullary differentiation and the delineation of adrenal glands, proximal ureter, renal arteries, and renal veins. For the functional imaging, the overall image quality was evaluated based on the corticomedullary differentiation and the renal edge sharpness. The highest value of 4 was defined to represent the best image quality, while a score of 1 indicated poor image quality.

Besides, for all the sequences, artifacts including B1 inhomogeneities, chemical shift, susceptibility, and motion artifacts were also graded on a four-point scale (4 = no image impairment, 3 = slight artifact, 2 = moderate artifact, 1 = non-diagnostic due to artifact).

### Quantitative image analysis

For the anatomical imaging, two experienced radiologists (C.Y. and M.Z.) consensually placed ROIs on the liver-, spleen-, renal cortex-, and muscle tissue. To minimize spatial variation effects, the ROIs were of identical size and placed at identical positions in all images avoiding the inclusion of confounding structures like blood vessels. Similar to prior comparison study [16], the contrast ratio (CR) was calculated as

1 $${\text{CR}} = \left( {\left| {{\text{Signal}}_{{{\text{liver}}}} - {\text{Signal}}_{{{\text{tissue}}}} } \right|} \right)/\left( {{\text{Signal}}_{{{\text{liver}}}} + {\text{Signal}}_{{{\text{tissue}}}} } \right).$$

Based on these four ROIs, SNR was calculated by the ratio of the mean signal value and the standard deviation (SD) of the background.

For the functional imaging, the ROIs were placed on the cortex and medulla separately. SNRs for cortex and medulla were measured on DWI images (*b* = 800 s/mm^2^) and T2* map. Furthermore, CNR of cortex/medulla was calculated as

2 $${\text{CNR}} = ({\text{Signal}}_{{{\text{cortex}}}} - {\text{Signal}}_{{{\text{medulla}}}} )/{\text{noise}}$$

For all the measurements, image noise was defined as the shared SD out of the three ROI measurements outside the body.

For both 3 T and 5 T renal DWI, the apparent diffusion coefficients (ADCs) were calculated from two b-values (*b* = 0, 800 s/mm^2^) by using a commercial workstation (uWS-MR, United Imaging Healthcare). The voxel intensity is given by

3 $$S = S_{0} \exp \left[ { - b{\text{ADC}}} \right]$$

where ${S}_{0}$ is the signal intensity without diffusion weighting and $b$ is the diffusion-sensitizing factor. T2* maps were generated using a log-linear, least squares method to fit the echo intensities pixel-by-pixel on the uWS-MR work station.

### Statistical analysis

The degree of agreement between the two radiologists at qualitative analysis was assessed with the kappa statistic (0–0.2, slight agreement; 0.21–0.4, fair agreement; 0.41–0.60, moderate agreement; 0.61–0.8, substantial agreement; > 0.8, excellent agreement). Wilcoxon signed rank-sum test was used to compare the visual evaluation scores and quantitative measurements between 3 and 5 T MRI. The Wilcoxon signed rank-sum test was also used to compare the individual cortical and medullary T2* and ADC of all the subjects. The Bonferroni correction was used to adapt the multiple tests. *p*-value < 0.05 was considered to represent statistically significant differences.

## Results

From January 2022 to July 2022, a cohort of 40 healthy volunteers (27 male and 13 female subjects; age, 42.6 ± 8.1 years) was finally included in this study. Two subjects were excluded because of abnormal findings of the kidney on MRI (*n* = 1, renal cyst) and incomplete MRI examination (*n* = 1).

All examinations at 5 T were performed successfully and were well tolerated by the subjects without any side effects. For all the sequences, interobserver agreement was substantial to excellent in image quality score and artifacts score (*κ* > 0.7). Since image quality scores and artifacts scores of all the raters were comparable, the mean scores of all the raters were used.

### Breath-hold T1-weighted QUICK 3D sequence

**Fig. 1:**
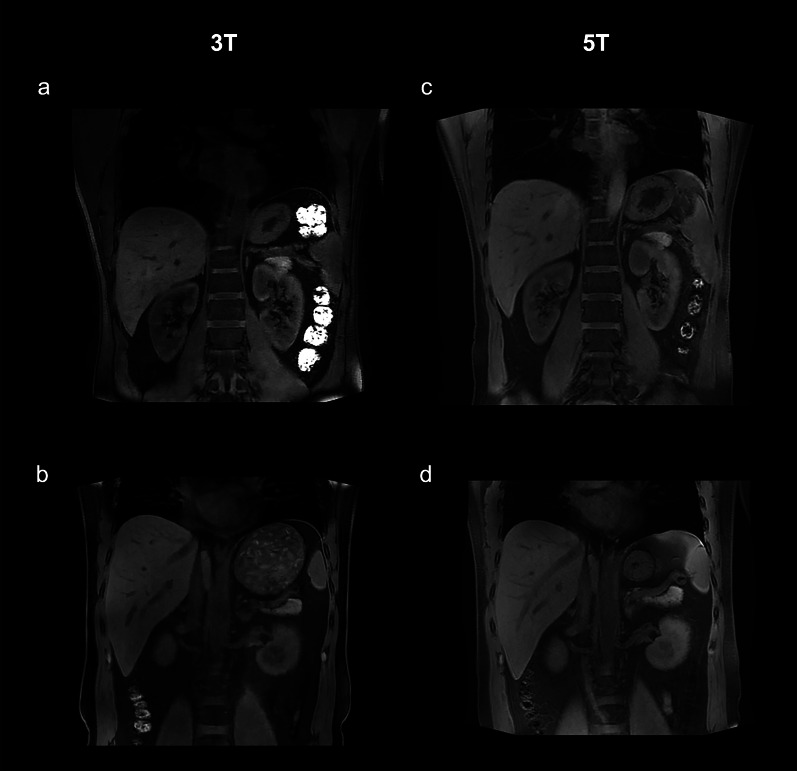
T1-weighted images of a 46-year-old male. **a**, **b** Coronal, breath-hold T1-weighted gradient-echo (GRE) sequence at 3 T; **c**, **d** Coronal, breath-hold T1-weighted GRE sequence at 5 T

Table 3 summarizes the qualitative image analysis results for anatomical imaging, and Fig. 1 shows representative images of coronal T1-weighted GRE sequence at 3 T and 5 T. For Breath-hold T1-weighted QUICK 3D sequence in the coronal plane, the overall image quality at 5 T (mean score 2.79) was significantly better than at 3 T (mean score 2.70) with significantly higher conspicuity of the renal arteries (mean score 3.23 for 3 T, mean score 3.39 for 5 T, *p* = 0.022) and veins (mean score 2.96 for 3 T, mean score 3.14 for 5 T, *p* = 0.016). The presence of artifacts for the 3 T MRI was not statistically significantly different from the 5 T MRI (mean score 3.25 for 3 T, mean score 3.21 for 5 T, *p* = 0.317). The same results were obtained from breath-hold T1-weighted QUICK 3D sequence in the axial plane (overall image quality: mean score 2.56 for 3 T, mean score 2.72 for 5 T, *p* = 0.001; presence of artifacts: mean score 3.24 for 3 T, mean score 3.18 for 5 T, *p* = 0.166).

**Table 3:** Average scores of qualitative image analysis for anatomical imaging at 3 T and 5 T

Parameters	Sequences					
	Axial T1W 3D GRE		Coronal T1W 3D GRE		Axial T2W FSE	
	3 T	5 T	3 T	5 T	3 T	5 T
Corticomedullary differentiation	3.31	3.29	3.26	3.18	2.55	2.61
*p*-value	0.317		0.149		0.540	
Adrenal gland	1.75	1.89	1.76	1.68	2.04	2.18
*p*-value	0.071		0.335		0.087	
Proximal ureter	2.10	2.18	2.35	2.50	2.54	2.44
*p*-value	0.214		0.080		0.129	
Renal artery	2.88	3.26	3.23	3.39	2.06	2.15
*p*-value	0.001		0.022		0.066	
Renal vein	2.74	2.96	2.96	3.14	1.94	2.01
*p*-value	0.008		0.016		0.098	
Overall image quality	2.56	2.72	2.70	2.79	2.23	2.28
*p*-value	0.001		0.022		0.059	

*T1W* T1-weighted; *T2W* T2-weighted; *GRE* gradient-echo; *FSE* fast spin echo

The results of the quantitative evaluation are summarized in Table 4. Compared to 3 T images, 5 T images showed significantly higher SNR and CR for all analyzed tissues in T1-weighted sequences (*p* < 0.05 for all the comparisons).

**Table 4:** Quantitative image analysis results for anatomical imaging at 3 T and 5 T

Parameters	Sequences					
	Axial T1W 3D GRE		Coronal T1W 3D GRE		Axial T2W FSE	
	3 T	5 T	3 T	5 T	3 T	5 T
SNR (renal cortex)	202.84 ± 58.97	310.50 ± 81.73	137.40 ± 63.79	205.78 ± 75.48	434.80 ± 127.36	799.59 ± 273.45
*p*-value	0.001		0.001		0.001	
SNR (liver)	239.68 ± 116.18	347.93 ± 137.50	155.65 ± 97.15	232.07 ± 118.94	138.86 ± 37.23	162.70 ± 54.00
*p*-value	0.001		0.001		0.013	
SNR (spleen)	204.83 ± 74.09	303.21 ± 63.46	127.81 ± 56.33	200.41 ± 66.96	329.66 ± 105.46	481.17 ± 172.74
*p*-value	0.001		0.001		0.001	
SNR (muscle)	206.97 ± 72.96	310.57 ± 57.64	129.70 ± 72.02	190.85 ± 75.09	151.13 ± 49.83	319.40 ± 85.73
*p*-value	0.001		0.005		0.001	
CR (renal cortex/liver)	0.10 ± 0.07	0.15 ± 0.07	0.08 ± 0.06	0.11 ± 0.06	0.51 ± 0.05	0.66 ± 0.08
*p*-value	0.026		0.013		0.001	
CR (spleen/liver)	0.07 ± 0.06	0.09 ± 0.08	0.08 ± 0.07	0.10 ± 0.07	0.39 ± 0.09	0.49 ± 0.05
*p*-value	0.030		0.016		0.013	
CR (muscle/liver)	0.07 ± 0.06	0.10 ± 0.07	0.08 ± 0.06	0.11 ± 0.07	0.12 ± 0.08	0.33 ± 0.12
*p*-value	0.030		0.009		0.002	

SNRs and CRs are given as mean ± standard deviation

*T1W* T1-weighted; *T2W* T2-weighted; *GRE* gradient-echo; *FSE* fast spin echo; *SNR* signal-to-noise ratio; *CR* contrast ratio

### T2-weighted fat-saturated FSE sequence with respiratory trigger

**Fig. 2:**
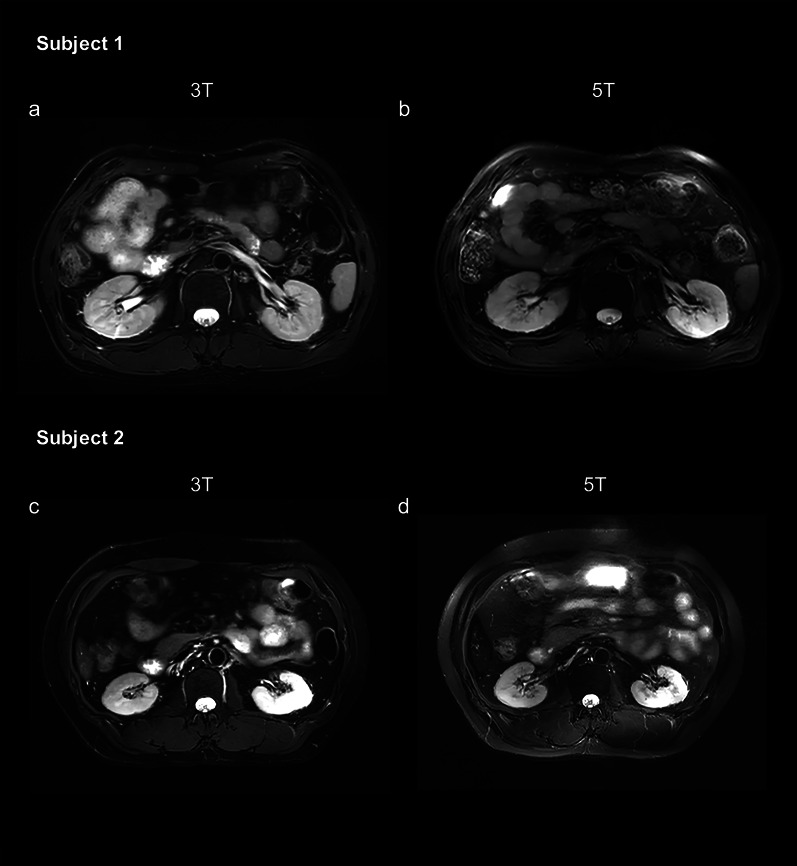
Axial, T2-weighted fast spin echo (FSE) sequence at 3 T (**a** and **c**) and 5 T (**b** and **d**) from subject 1 (49-year-old male) and subject 2 (40-year-old male). For subject 2, T2-weighted FSE at 5 T exhibited a higher level of artifacts compared to the 3 T examination

Figure 2 shows representative images of the T2-weighted FSE sequence at 3 T and 5 T. For the T2-weighted FSE sequence, 5 T MRI showed comparable overall image quality to 3 T MRI (mean score 2.23 for 3 T, mean score 2.28 for 5 T, *p* = 0.059), while a significantly higher level of artifacts was observed at 5 T (mean score 3.21 for 3 T, mean score 3.03 for 5 T, *p* = 0.002). As shown in Table 4, compared to 3 T images, 5 T images showed significantly higher SNR and CR for all analyzed tissues (*p* < 0.05 for all the comparisons).

### Functional MRI

**Fig. 3:**
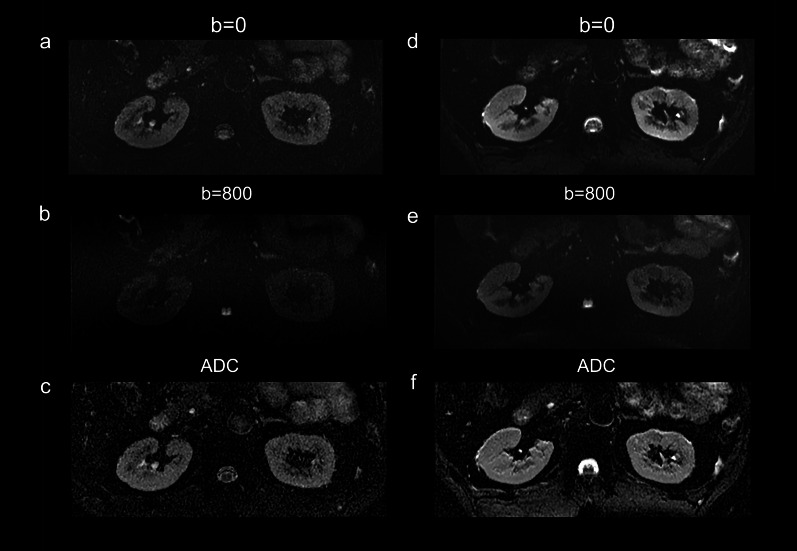
Diffusion-weighted images and corresponding apparent diffusion coefficient map at 3 T (**a** and **c**) and 5 T (**d** and **f**) from a 42-year-old female

**Fig. 4:**
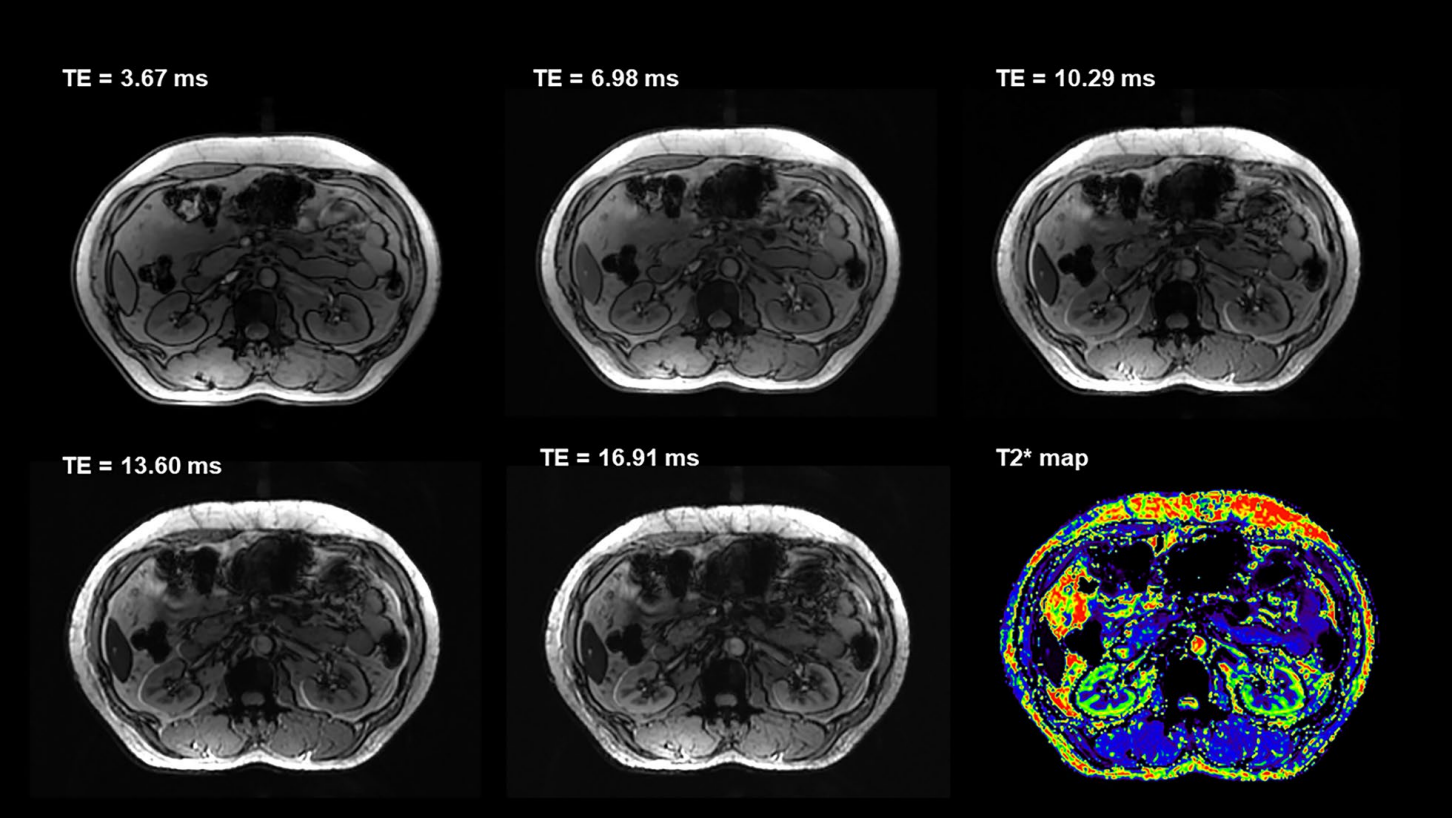
Axial, multi-echo gradient-echo T2* mapping at 5 T from a 56-year-old female

For all the functional MRI sequences (Figs. 3 and 4), including renal EPI-DWI sequence (overall image quality: mean score 2.99 for 3 T, mean score 3.29 for 5 T, *p* < 0.001; corticomedullary differentiation: mean score 2.89 for 3 T, mean score 3.31 for 5 T, *p* < 0.001; renal edge sharpness: mean score 3.09 for 3 T, mean score 3.26 for 5 T, *p* = 0.007) and T2* mapping (overall image quality: mean score 3.15 for 3 T, mean score 3.48 for 5 T, *p* < 0.001; corticomedullary differentiation: mean score 3.13 for 3 T, mean score 3.54 for 5 T, *p* < 0.001; renal edge sharpness: mean score 3.18 for 3 T, mean score 3.41 for 5 T, *p* < 0.001), the image quality of 5 T images was significantly better than 3 T images, while there is no significant difference between the presence of artifacts (DWI: mean score 3.14 for 3 T, mean score 3.10 for 5 T, *p* = 0.083; T2* mapping: mean score 3.06 for 3 T, mean score 3.03 for 5 T, *p* = 0.439).

Compared to 3 T, SNR of the renal cortex at 5 T was approximately 60% and 20% higher for DWI and T2* mapping, respectively (Table 5). In the renal medulla, SNR increases from 3 to 5 T were approximately 65% (DWI) and 15% (T2* mapping). CNR of cortex/medulla at 5 T was approximately 80% (DWI) and 30% (T2* mapping) higher compared to 3 T. All differences reached statistical significance.

**Table 5:** Quantitative image analysis results for functional imaging at 3 T and 5 T

Parameters	Sequences			
	DWI (*b* = 800 s/mm^2^)		T2* map	
	3 T	5 T	3 T	5 T
SNR (cortex)	22.04 ± 5.42	36.13 ± 8.92	24.93 ± 5.11	30.21 ± 5.18
*p*-value	0.001		0.001	
SNR (medulla)	14.68 ± 4.45	24.28 ± 6.78	14.87 ± 4.20	17.08 ± 4.03
*p*-value	0.001		0.036	
CNR (cortex/medulla)	6.60 ± 2.30	11.86 ± 5.48	10.06 ± 3.39	13.12 ± 3.57
*p*-value	0.001		0.017	

SNRs and CNRs are given as mean ± standard deviation

*DWI* diffusion-weighted imaging; *SNR* signal-to-noise ratio; *CNR* contrast-to-noise ratio

ADC of the cortex was significantly higher than that of the medulla at both 3 T and 5 T (*p* = 0.018 for both 3 T and 5 T). Further, there was no significant difference between measured ADC values at 3 T and 5 T for both cortex (1.92 × 10^−3^ mm^2^/s at 3 T and 1.96 × 10^−3^ mm^2^/s at 5 T, *p* = 0.176) and medulla (1.38 × 10^−3^ mm^2^/s at 3 T and 1.41 × 10^−3^ mm^2^/s at 5 T, *p* = 0.398).

T2* value of the cortex was significantly higher than that of the medulla at both 3 T and 5 T (*p* = 0.001 for both 3 T and 5 T). Compared to 3 T, renal MRI at 5 T resulted in significantly shorter T2* values in both the cortex (69.33 ms at 3 T and 46.53 ms at 5 T, *p* < 0.001) and medulla (28.92 ms at 3 T and 16.29 ms at 5 T,* P* < 0.001).

## Discussion

In this study, concerning T1-weighted sequences, 5 T MRI demonstrated better overall image quality with higher conspicuity of the renal veins and arteries than 3 T MRI. The presence of artifacts was comparable between 3 and 5 T on T1-weighted sequences. For the T2-weighted FSE sequence, 5 T MRI showed comparable overall image quality to 3 T MRI, while a significantly higher level of artifacts was observed at 5 T. Furthermore, 5 T MRI contributed to higher SNR and CR for abdomen organ tissues, including liver, spleen, and kidneys. Prior studies stated that these higher SNRs enable an improvement in the evaluation of anatomical details as well as increased accuracy for the depiction of pathological findings [7, 17, 18]. For functional imaging at 5 T, SNR of cortex and medulla and CNR of cortex/medulla were significantly higher than those at 3 T, leading to improved corticomedullary discrimination. There was no significant difference between measured ADC values at 3 T and 5 T for both cortex and medulla. T2* relaxation times decreased with the increase of magnetic field strength.

Despite the lack of intravenous contrast agent, the inherently high signal intensity of the non-enhanced vasculature in the T1-weighted sequence at 5 T provided super conspicuity of the renal vasculature, which is confirmed by previous 7 T T1-weighted MRI investigations [19, 20]. The T1 times of surrounding stationary tissue are prolonged by 10–20% compared with blood [21]. Hence, due to the decreased relaxation rate, fast repetitions of RF excitation pulses result in improved background signal suppression of static tissues associated with improved vessel-to-background contrast. This potential for robust vascular imaging without contrast agent is attractive because of the lower cost and data linking nephrogenic systemic fibrosis to gadolinium contrast agent exposure [22]. Furthermore, the prior study revealed that T1-weighted sequences at 7 T MRI showed significantly higher impairment due to the presence of artifacts, compared to 3 T MRI [12]. However, in this study, no significant differences were observed for T1-weighted sequences in the presence of artifacts between 3 and 5 T MRI. The possible explanation is that motion artifacts are not substantially reinforced at higher field strength and that factors such as patient compliance, positioning, and fixation are crucial [23]. In addition, the B1 inhomogeneity and susceptibility artifacts were more pronounced at 7 T MRI than at 5 T MRI. Therefore, T1-weighted sequences at 5 T have potential to be utilized in clinical settings.

T2-weighted MR sequences are considered as a significant tool to characterize abdominal lesions [24, 25]. However, the T2-weighted FSE sequence for abdominal imaging at ultra-high field MRI remains challenging and is susceptible to be strongly impaired because of signal heterogeneities [13, 26]. SAR restrictions on the one hand and the limited available RF peak power available for achieving the large flip angles needed for spin echo imaging on the other hand are unsolved challenges [27]. Though recent methodological developments regarding transmit strategies and SAR supervision enabled the exploitation of the potential of whole-body imaging at ultra-high field MRI, few studies reported the investigations about T2-weighted abdominal imaging [13, 28]. Our results, in accordance with previous comparison studies, showed that susceptibility artifacts and B1 inhomogeneities of T2-weighted images were significantly increased as compared to lower field strengths [12, 29]. Nevertheless, the image quality of T2-weighted images at 5 T was scored as high as for 3 T. The possible explanation is that the overall image quality of the T2-weighted FSE sequence was evaluated based on the corticomedullary differentiation and the delineation of adrenal glands, proximal ureter, renal arteries, and renal veins. Besides, though a significantly higher level of artifacts was observed at 5 T, the numbers of scores were not substantially different (mean score 3.21 for 3 T, mean score 3.03 for 5 T) and the artifacts at 5 T were no to slight impairment without substantial effect on the image quality.

DWI is a powerful method that has been used to evaluate a variety of renal pathologies, including focal lesions, acute and chronic disease, and allografts [30–32]. Critical shortcomings of EPI-DWI image quality are susceptibility-related artifacts that scale linearly with the magnetic field strength [33]. To minimize these artifacts, several acquisition methods, including reduced FOV acquisition, have been proposed which aim for a reduction of the echo train length [34]. In this study, for high-resolution renal EPI-DWI sequence with reduced FOV, the image quality of 5 T images was significantly higher than 3 T images, while there was no significant difference in the level of artifacts. ADC of the cortex is significantly higher than that of the medulla at both 3 T and 5 T, which is supported by prior studies [30, 35]. Moreover, our results demonstrated that there was no significant difference between measured ADC values at 3 T and 5 T for both cortex and medulla. Theoretically, ADC is independent of the magnetic field strength [36]. According to previous studies, the effect on ADC measurements is of minor importance for 1.5- and 3.0-T MR systems from the same vendor, and ADC values of renal cortex and medulla were in the same range for both field strengths [37, 38].

Renal tissue hypoxia plays an important role in the pathophysiology of acute kidney injury and its progression to chronic kidney disease [39]. The pathophysiology of diabetic nephropathy is also thought to be heavily influenced by the disturbance of the equilibrium between renal oxygen supply and demand [40]. Thus, in vivo assessment of renal tissue oxygenation is crucial. As the increased concentration of deoxyhemoglobin contributes to shorter T2* [41], T2*-weighted map obtained by multi-echo GRE sequence could be used to depict the oxygenation level within the kidneys in both healthy subjects and patients with renal diseases. The benefits of this blood oxygen level-dependent (BOLD) MRI technique at higher magnetic field strength (> 3 T) are potentially large because its sensitivity to detect differences in oxygenation improves at higher fields [41]. Theoretically, T2* and R2* magnitude is scaled by magnetic field strength [42]. In accordance with previous studies [43], our results revealed that T2* relaxation times in both cortex and medulla significantly decreased with the increase of magnetic field strength.

This study had several limitations. First, our study population was relatively small and consisted entirely of healthy subjects. Further research with a large number of subjects and patients with abdominal diseases is necessary. Second, in this study, the protocols of DWI and T2* mapping used were only dedicated to assess renal parenchyma. Protocols for the evaluation of other abdomen organs, especially the liver, need to be developed. Third, the spatial coverage of the imaging sequences in this initial study is limited. Other functional imaging sequences, such as dynamic contrast-enhanced (DCE) and arterial spin labeling (ASL) sequences, could be the focus of future studies.

## Conclusions

In summary, this initial study indicated that 5 T MRI provides anatomical and functional images of the abdomen with image quality comparable to or better than 3 T. T1-weighted sequence at 5 T demonstrated higher conspicuity of the renal veins and arteries. Besides, for DWI and T2* mapping, 5 T MRI contributed to improved corticomedullar discrimination than 3 T MRI. Further optimization of sequences and RF technology can be expected to enable the acquisition of better image quality with corresponding clinical diagnostic value.

## Data Availability

The datasets used and/or analyzed during the current study are available from the corresponding author on reasonable request.

## Abbreviations

ADC: Apparent diffusion coefficient
CNR: Contrast-to-noise ratio
CR: Contrast ratio
DWI: Diffusion-weighted imaging
EPI: Echo-planar imaging
FA: Flip angle
FOV: Field-of-view
FSE: Fast spin echo
GRE: Gradient-echo
MRA: Magnetic resonance angiography
MRI: Magnetic resonance imaging
RF: Radiofrequency
SAR: Specific absorption rate
SNR: Signal-to-noise ratio
TE: Echo time
TR: Repetition time
